# BALB/c mice genetically susceptible to proteoglycan-induced arthritis and spondylitis show colony-dependent differences in disease penetrance

**DOI:** 10.1186/ar2613

**Published:** 2009-02-16

**Authors:** Balint Farkas, Ferenc Boldizsar, Oktavia Tarjanyi, Anna Laszlo, Simon M Lin, Gabor Hutas, Beata Tryniszewska, Aaron Mangold, Gyorgy Nagyeri, Holly L Rosenzweig, Alison Finnegan, Katalin Mikecz, Tibor T Glant

**Affiliations:** 1 Section of Molecular Medicine, Department of Orthopedic Surgery, Rush University Medical Center, 1735 W. Harrison Street, Cohn Research Building, Chicago, IL 60612, USA; 2 Department of Immunology and Biotechnology, University of Pecs, Ifjusag u. 13, Pecs, Hungary; 3 Biomedical Informatics Center, Northwestern University, 750 N. Lake Shore Drive, Chicago, IL 60611, USA; 4 Department of Ophthalmology, Portland, Oregon Health Science University, 3181 S.W. Sam Jackson Park Road, Portland, OR 97239, USA; 5 Department of Internal Medicine (Section of Rheumatology), Rush University Medical Center, 1730 W. Harrison Street, Cohn Research Building, Chicago, IL 60612, USA; 6 Department of Immunology/Microbiology, Rush University Medical Center, 1730 W. Harrison Street, Cohn Research Building, Chicago, IL 60612, USA; 7 Department of Biochemistry, Rush University Medical Center, 1730 W. Harrison Street, Cohn Research Building, Chicago, IL 60612, USA

## Abstract

**Introduction:**

The major histocompatibility complex (H-2d) and non-major histocompatibility complex genetic backgrounds make the BALB/c strain highly susceptible to inflammatory arthritis and spondylitis. Although different BALB/c colonies develop proteoglycan-induced arthritis and proteoglycan-induced spondylitis in response to immunization with human cartilage proteoglycan, they show significant differences in disease penetrance despite being maintained by the same vendor at either the same or a different location.

**Methods:**

BALB/c female mice (24 to 26 weeks old after 4 weeks of acclimatization) were immunized with a suboptimal dose of cartilage proteoglycan to explore even minute differences among 11 subcolonies purchased from five different vendors. *In vitro*-measured T-cell responses, and serum cytokines and (auto)antibodies were correlated with arthritis (and spondylitis) phenotypic scores. cDNA microarrays were also performed using spleen cells of naïve and immunized BALB/cJ and BALB/cByJ mice (both colonies from The Jackson Laboratory, Bar Harbor, ME, USA), which represent the two major BALB/c sublines.

**Results:**

The 11 BALB/c colonies could be separated into high (n = 3), average (n = 6), and low (n = 2) responder groups based upon their arthritis scores. While the clinical phenotypes showed significant differences, only a few immune parameters correlated with clinical or histopathological abnormalities, and seemingly none of them affected differences found in altered clinical phenotypes (onset time, severity or incidence of arthritis, or severity and progression of spondylitis). Affymetrix assay (Affymetrix, Santa Clara, CA, USA) explored 77 differentially expressed genes (at a significant level, *P *< 0.05) between The Jackson Laboratory's BALB/cJ (original) and BALB/cByJ (transferred from the National Institutes of Health, Bethesda, MD, USA). Fourteen of the 77 differentially expressed genes had unknown function; 24 of 77 genes showed over twofold differences, and only 8 genes were induced by immunization, some in both colonies.

**Conclusions:**

Using different subcolonies of the BALB/c strain, we can detect significant differences in arthritis phenotypes, single-nucleotide polymorphisms (SNPs), and a large number of differentially expressed genes, even in non-immunized animals. A number of the known genes (and SNPs) are associated with immune responses and/or arthritis in this genetically arthritis-prone murine strain, and a number of genes of as-yet-unknown function may affect or modify clinical phenotypes of arthritis and/or spondylitis.

## Introduction

Rheumatoid arthritis (RA) is a chronic autoimmune disease that leads to inflammatory cartilage destruction and bone erosion in synovial joints. Although the pathological mechanism of RA is unknown, both environmental and genetic factors are thought to be involved in the etiology and pathogenesis of the disease [1]. Animal models, especially those that involve joint pathology in genetically altered rodents, are invaluable aids in the research of human autoimmune diseases [2-6]. Among the systemic animal models of RA, cartilage proteoglycan (PG) aggrecan-induced arthritis (PGIA) is a T cell-dependent and autoantibody/B cell-mediated disease in BALB/c mice which is frequently accompanied by spondylitis [7-10]. In addition to the major histocompatibility complex (MHC), PGIA and PG-induced spondylitis (PGIS) are controlled by multiple genetic loci [9,11]. Although various non-MHC genetic loci (quantitative trait loci; QTLs) may contribute to disease, different combinations of these QTLs may result in a remarkably uniform clinical phenotype of arthritis [12].

Due to a specific genetic background, the BALB/c strain shows a strong predisposition toward arthritis. In addition to PGIA, immunization with cartilage link protein [13] or human cartilage glycoprotein-39 (HC-gp39) [14] can induce arthritis, but only in BALB/c mice. Moreover, interleukin-1 (IL-1) receptor antagonist protein-deficient mice [15] and SKG mice, in which a spontaneous point mutation occurred in ZAP-70, develop spontaneous arthritis [16], both only in the BALB/c background.

Despite the efforts of companies to maintain genetically homogenous inbred colonies, there are differences among BALB/c colonies/substrains (for example, in body weight, size of littermates, and the composition of intestinal bacterial flora) maintained at different locations by the same vendor. According to the online public database of The Jackson Laboratory (Bar Harbor, ME, USA) [17], there are at least 492 single-nucleotide polymorphism (SNP) differences between their two inbred BALB/cJ and BALB/cByJ colonies; of these, at least 59 SNPs are present in 33 immune-regulatory genes in the mouse genome (F. Boldizsar and T.T. Glant, unpublished *in silico *analysis data). Some of these known, or as-yet-unknown, mutations may significantly influence the pathogenesis and progression of PGIA or PGIS.

Since we have 'simplified' the model by replacing the highly purified human fetal cartilage PG [7,18] with PG isolated from human osteoarthritic cartilage [19,20] and changed the Freund's adjuvants to a synthetic adjuvant [21], the PGIA/PGIS model became available to a wide range of applications, including the testing of new pharmacological agents. However, we and others observed differences in the onset, incidence, and severity of arthritis, even when the source of antigen (for example, our laboratory) and immunization protocols were the same. Therefore, either local environmental components or the source of BALB/c colony might account for the different levels of susceptibility to, or severity of, PGIA. Because environmental factors also play critical roles in RA susceptibility [22] and different BALB/c colonies may have different panels of spontaneous mutations, it has become necessary to test these components under uniform conditions. In the present study, we investigated the disease parameters (onset time, susceptibility, severity, and progression) simultaneously in various colonies of BALB/c mice in the same experimental setup. Because the BALB/c strain is highly susceptible to PGIA (and PGIS) and sooner or later all immunized animals develop arthritis independently of the colony source, we used a suboptimal dose of PG antigen to be able to monitor even minute differences among the colonies.

## Materials and methods

### Chemicals, antigen, animals, and immunization of mice with cartilage proteoglycan aggrecan

All chemicals, unless otherwise indicated, were purchased from Sigma-Aldrich (St. Louis, MO, USA) or Fischer Scientific Co. (Chicago, IL, USA). Mouse-specific cytokine enzyme-linked immunosorbent assay (ELISA) kits were purchased from R&D Systems (Minneapolis, MN, USA) or BD Biosciences (San Jose, CA, USA). Cartilage specimens from knee joints were obtained from osteoarthritic patients undergoing joint replacement surgery. The use of human cartilage for PG isolation was approved by the Institutional Review Board of Rush University Medical Center (RUMC) (Chicago, IL, USA). PG isolation has been described in detail [19,20]. All animal experiments were approved by the Institutional Animal Care and Use Committee of RUMC. Animals were maintained in a pathogen-free environment in the same room and rack. A total of 178 (retired breeder) female BALB/c mice of 11 colonies (Table 1) were ear-tagged, and registered mice (all 24 to 26 weeks old) were randomly mixed and left for acclimatization to the local environment for 4 weeks prior to the first immunization.

**Table 1:** Arthritis susceptibility, severity, and onset of different BALB/c colonies

Colony^a^		Vendors	Arthritic/total number of animals		Arthritis score (acute)^b^	Onset score^c^
				
Number	Symbol		Incidence	Percentage		
1	●	Portage P08; Charles River Laboratories, Inc. (Wilmington, MA, USA)	16/16	100%	11.0 ± 1.1	2.3 ± 0.3
2	●	Canada II; Charles River Laboratories, Inc.	15/15	100%	10.3 ± 1.3	2.5 ± 0.4
3	●	Harlan Laboratories, Inc. (Indianapolis, IN, USA)	12/13	92%	9.7 ± 1.5	2.8 ± 0.5
4	■	Kingston K72; Charles River Laboratories, Inc.	13/14	93%	8.8 ± 1.2	2.1 ± 0.4
5	■	Raleigh R02; Charles River Laboratories, Inc.	15/15	100%	7.6 ± 1.0	1.9 ± 0.3
6	■	Taconic Farms, Inc. (Hudson, NY, USA); Charles River Laboratories, Inc.	15/18	83%	7.5 ± 1.4	1.6 ± 0.3
7	■	NCI/Kingston (Charles River Laboratories, Inc.)	17/20	85%	6.8 ± 0.9	1.5 ± 0.2
8	■	BALB/cJ; The Jackson Laboratory (Bar Harbor, ME, USA)	16/19	84%	6.6 ± 1.1	2.1 ± 0.3
9	■	Raleigh R12; Charles River Laboratories, Inc.	10/13	77%	6.4 ± 1.5	1.2 ± 0.2
10	▲	Hollister H42; Charles River Laboratories, Inc.	10/15	67%	3.8 ± 0.8	1.1 ± 0.2
11	▲	Bailey's BALB/c ByJ; The Jackson Laboratory	15/20	75%	2.4 ± 0.7	1.0 ± 0.2

**Figure 1:**
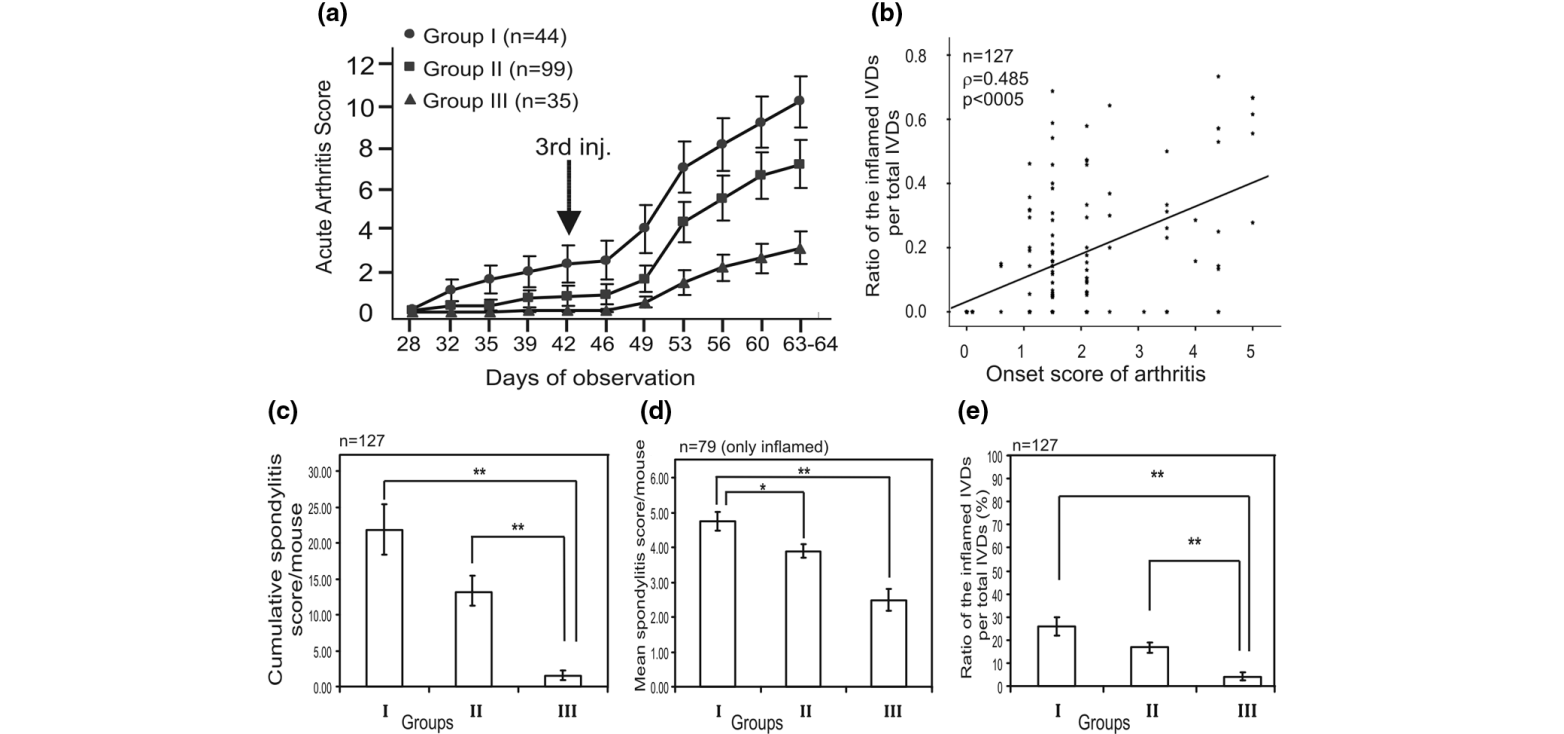
Progression and severity of arthritis in 11 BALB/c colonies sorted into three different groups (listed in Table 1), correlation between the onset of arthritis and spine involvement, and comparison of the three arthritic groups with different spine inflammation scores. **(a) **Each animal was scored for arthritis three times a week, and scores are shown as mean ± standard error of the mean. Arrow indicates the third injection, administrated on day 42. Significant differences (*P *< 0.01), calculated by one-way analysis of variance, were found from day 32 between groups I, II, and III. **(b) **The ratio of the number of inflamed intervertebral discs (IVDs) per the total number of IVDs showed positive significant correlation (Pearson correlation coefficient ρ = 0.485; *P *< 0.0005) with the onset of arthritis. **(c-e) **Significant differences were found among the three arthritic groups when compared with three spine representative scores: cumulative spondylitis score (c), the mean spondylitis score (d), and the ratio of the number of inflamed IVDs per total number of IVDs (e). Asterisks indicate the level of significance between groups (*P < 0.05 and **P < 0.01) using Tamhane (c, e) (n = 127) and least significant difference (d) (n = 79) *post hoc *tests.

All animals were immunized with human cartilage proteoglycan aggrecan in dimethyldioctadecyl-ammonium bromide. Values represent mean ± standard error of the mean. ^a^Colony numbers indicate the different BALB/c colonies. ^b^Highest arthritis score measured at any time point of the experiment. ^c^Onset score was calculated at the end of the experiment (days 63 and 64) and ranged from the earliest onset of inflammation (5) to no arthritis (0). Circles (●) represent the most arthritis-prone colonies. Designation was based on the statistical analysis showing no significant differences between these three colonies (1 to 3) comparing two major clinical variables: onset and severity. Therefore, these three colonies were combined and designated as group I (Figure 1a). Squares (■) indicate the average clinical phenotype of arthritis (colonies 4 to 9) with no significant differences using onset and severity scores as clinical phenotype markers. This combined group is designated as group II (Figure 1a). Triangles (▲) represent the two least arthritic or least susceptible colonies, 10 and 11. Data of these two colonies were combined, and the two colonies together were designated as group III (Figure 1a). NCI, National Cancer Institute (Bethesda, MD, USA).

Mice were injected intraperitoneally with a 'suboptimal' dose of human cartilage PG aggrecan (equivalent to 75 μg, instead of the 'standard' dose of 100 μg of core protein of PG) emulsified with 2 mg of dimethyldioctadecyl-ammonium bromide (DDA) adjuvant in 200 μL of phosphate-buffered saline (pH 7.4). DDA is a synthetic adjuvant with positively charged micelle-forming hydrophobic-hydrophilic (detergent-like) properties and does not contain mineral oil or mycobacterial components as Freund's adjuvants do [21]. Intraperitoneal injections were given on days 0, 21, and 42 of the experiment, and mice were sacrificed on days 63 and 64. The goal of using a suboptimal dose of cartilage PG aggrecan was necessary; otherwise, all animals develop arthritis with a high arthritis score after the third PG aggrecan (100 μg) injection in DDA [20,21]. About half a year later, these experiments were repeated using BALB/cJ and BALB/cByJ strains (The Jackson Laboratory) and Portage P08 and Hollister H42 (Charles River Laboratories, Inc., Wilmington, MA, USA) as well as National Cancer Institute (NCI) (Bethesda, MD, USA) (Kingston) BALB/c colonies (10 to 15 animals per group).

### Clinical and histological assessment of arthritis and spondylitis

Arthritis severity was determined using a visual scoring system based on the degree of swelling and redness of the front and hind paws [7,18,20]. Animals were examined at least three times a week and inflammation was scored from 0 to 4 for each paw, resulting in a cumulative arthritis score ranging from 0 to 16 for each animal [7,20]. To monitor early inflammatory reactions as well, in this particular study, an acute arthritis score of 0.5 was given if at least two interphalangeal, metacarpo-phalangeal, or metatarso-phalangeal joints were swollen but the paw inflammation (swelling and redness) did not reach the 'standard' level of an arthritis score of 1 [20]. Animals were scored alternatively by two investigators in a blind manner. Incidence of the disease was expressed as the percentage of arthritic mice to the total number of PG-immunized mice per colony. Acute arthritis (severity) score was applied only to arthritic animals. In addition, an arbitrary score (from 5 to 0) from the earliest onset of arthritis (onset score of 5) to negative (onset score of 0) was established for each mouse [23,24]. This onset score represents how quickly a mouse developed arthritis.

Upon sacrifice, limbs and spines were removed, fixed in 10% formalin, acid-decalcified, and processed in accordance with standard histological procedures [7-9,20]. A total of 2,298 intervertebral discs (IVDs) of 127 spines (7 to 15 per colony) were examined and scored. A modified histological scoring system of the spine [10] was established by assessment of the severity of spine involvement, which may achieve a score for each IVD, ranging from 0 to 8. No inflammation was scored as 0, inflammatory (leukocyte) cell accumulation (peridiscitis and enthesitis) was scored as 1 or 2, progression of IVD resorption was scored as 3 to 6, fibrotic or fibro-cartilaginous ankylosis (with complete disc resorption) received a score of 7, and complete ankylosis due to chondrophyte/osteophyte formation was scored as 8. A cumulative spodyloarthropathy score (the sum of spondylitis scores per spine) was calculated for each animal.

### Measurements of serum cytokines and anti-proteoglycan antibodies and the lymphocyte responses

Serum cytokines IL-1β, IL-4, IL-6, interferon-gamma (IFN-γ), and tumor necrosis factor-alpha were measured by ELISA. Antigen-specific lymphocyte responses were measured in spleen cell cultures in the presence or absence of 50 μg/mL human PG antigen. Antigen-specific IL-2 production was measured as a proliferative response of CTLL-2 cells to IL-2 in 48-hour spleen cell supernatants (CTLL-2 bioassay) [20]. Lymphocyte proliferation was assessed on day 5 of culture by measuring [^3^H]-thymidine incorporation [18,20], and antigen-specific T-cell proliferation was expressed as stimulation index [7,18,20]. *In vitro *production of the above-listed cytokines was also measured in supernatants of antigen (PG)-stimulated (50 μg/mL) spleen cell cultures on day 5 using ELISA. Secreted cytokine concentrations were normalized to nanograms per million cells [9,11].

PG-specific serum antibodies were measured by ELISA using at least three different serum dilutions. Highly purified human or mouse cartilage PG [25] was immobilized onto the surface of Nunc-Maxisorp 96-well plates (Nalge Nunc, Naperville, IL, USA) [20]. For PG-specific IgG isotype assays, peroxidase-labeled goat anti-mouse IgG1 (Zymed Laboratories, Inc., now part of Invitrogen Corporation, Carlsbad, CA, USA) and IgG2a (BD Biosciences) were employed. Serum PG-specific antibody levels were calculated using serial dilutions of pooled and standardized sera of mice with PGIA [20].

### Affymetrix hybridization and related statistical analysis

RNA samples from spleen cells of naïve and immunized (12 days after the intraperitoneal PG injection) mice were extracted with TriReagent (Sigma-Aldrich) in accordance with the instructions of the manufacturer. Affymetrix hybridization (Affymetrix, Santa Clara, CA, USA) was performed using 'Mouse Genome 430 2.0' gene chips. Biotinylation of cRNA, labeling, and hybridization were processed by the Genomic Core Facility of the University of Illinois at Chicago. Data were analyzed using the GeneSpring GX 10.0 software package (Agilent Technologies, Inc., Santa Clara, CA, USA). Robust multi-array average [26] summarization algorithm (with quantile normalization and median polish probe summarization procedures) and baseline transformation (that is, per gene normalization; baseline to median of all 12 samples) were run on data using a logarithmic scale. All sample replications passed quality control. For pairwise comparisons, the Student-Newman-Keuls *post hoc *test was performed after one-way analysis of variance on four groups (naïve BALB/cJ, naïve BALB/cByJ, immunized BALB/cJ, and immunized BALB/cByJ) to identify statistically significant (*P *< 0.05) differentially expressed transcripts and statistical differences between naïve and immunized mice of the two colonies. Asymptotic *P *value computation and Benjamini-Hochberg false discovery rate multiple testing correction were applied [27]. Hierarchical clustering was applied to significantly differentially expressed genes, based on the Pearson centered distance metric and centroid linkage rule. Differentially expressed transcripts were annotated with the GeneSpring software. The bivariate linear correlation (Pearson) test was performed to identify statistical correlations among spine and arthritis parameters. The Fisher exact chi-square test was applied when normal and diseased animals were compared. These statistical analyses were performed using SPSS version 16.0 statistical software (SPSS Inc., Chicago, IL, USA).

## Results

### Susceptibility, severity, and onset of arthritis in different BALB/c colonies

Based on the visual scoring system [20] and later confirmed by histology, we could sort the 11 BALB/c colonies into three major groups. There were no statistical differences in arthritis severity and onset time within any of the three groups (Table 1 and Figure 1a). Overall, arthritis scores ranged from 2.4 ± 0.7 to 11.0 ± 1.1 and the onset score of arthritis ranged from 1.0 ± 0.2 to 2.8 ± 0.5 (Table 1). Most of the BALB/c colonies (colony numbers 4 to 9, henceforth called group II) developed arthritis at an average severity of 7.2 ± 0.5 and at onset scores of 1.8 ± 0.1 (Table 1 and Figure 1a). Compared with group II, group I (colonies 1 to 3, Table 1) comprised the most susceptible substrains, which developed arthritis earlier and with greater severity than any other colonies. In contrast, group III (colonies 10 and 11, Table 1) showed the least severe arthritis (mean arthritis score of 3.0 ± 0.6) with delayed onset time (1.0 ± 0.2), and approximately 30% of the immunized animals did not have arthritis at the end of the experiment (Table 1 and Figure 1a). In arthritic animals, the histopathological abnormalities (cellular infiltration, synovitis, pannus formation, and cartilage and bone erosions) were similar to those (data not shown) described earlier in numerous papers [7-9,20,28], and there were no differences in the histopathology when peripheral joints of any subcolonies with the same clinical scores [20] were compared (data not shown).

### Histopathology of the spine

A total of 127 spines were formalin-fixed, x-ray-imaged, and then processed for histological analysis. Following the earlier scoring system [10], IVD involvement was analyzed using three parameters: (a) the cumulative spondyloarthropathy score of each animal (Figure 1c), (b) the mean (IVD) inflammatory score per animal (Figure 1d), and (c) the ratio of the number of the inflamed IVDs per total number of IVDs (expressed as a percentage) (Figure 1e). In the scoring of the 127 spine sections, spondylitis was diagnosed in 62.2% of BALB/c mice, which was significantly lower (*P *< 0.05) than the mean of arthritis incidence (86.5%; n = 178). This observation confirmed that arthritis and spondylitis could occur either together or separately in BALB/c mice [9] and that, most likely, different genes of different QTLs control PGIA and PGIS [9,29]. However, the most susceptible and most severely arthritic BALB/c colonies (Table 1) showed the most extensive spine involvement (Figure 1c–e) as assessed by using any of the three spondylitis parameters listed above. Similarly, animals that developed arthritis sooner exhibited more progressive spondylitis (Figure 1b). Although no PGIS-resistant BALB/c colony was found, there were large individual variations in the spine involvement. In addition, neighboring IVDs of the same animal frequently showed different stages of inflammation. Typically, the most affected spine segments were the distal lumbar and distal cervical regions, whereas the IVDs in the thoracic and proximal lumbar regions remained less affected.

### T cell- and B cell-mediated immune responses

**Figure 2:**
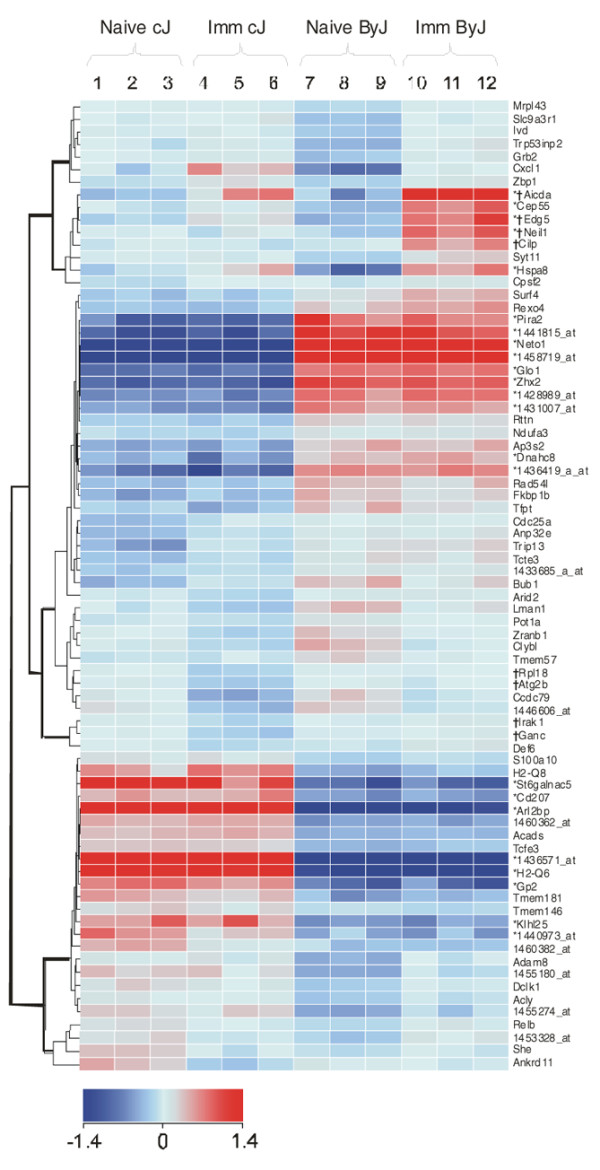
Hierarchical clusterization comparing the 77 genes expressed differently at significant levels in spleen cells of naive and proteoglycan-immunized (not-yet-arthritic) mice of BALB/cJ and BALB/cByJ colonies (n = 3 of each, four-group cross-comparison: naïve BALB/cJ versus immunized BALB/cJ, naïve BALB/cByJ versus immunized BALB/cByJ, naïve BALB/cJ versus naïve BALB/cByJ, and immunized BALB/cJ versus immunized BALB/cByJ). Color code indicates the normalized intensity expression values (with baseline transformation) on a logarithmic scale. Twenty-three genes showing over twofold differences in any of the four comparisons are labeled with asterisks. Whenever a gene name was not identified (n = 14), the original probe set ID (number_at), the Riken ID (numberRik), or the expressed sequence tag clone number is used. Those genes that showed significant differences only in response to immunization (n = 8) are labeled with the '†' symbol. Original data files are available via Gene Expression Omnibus (accession number [GEO:GSE13730] and National Center for Biotechnology Information tracking system number 15549466).

Despite screening a wide spectrum of immunological parameters, we could not identify 'colony-specific' cytokine, T-cell, or B-cell responses. *In vitro *tests (T-cell proliferation and cytokine production) showed evidence of T-cell activation in response to PG stimulation, but T cell responses did not correlate significantly with either arthritis or spondylitis scores. Interestingly, female BALB/c mice of the Hollister and ByJ colonies (Table 1, colonies 10 and 11, group III), which were the animals least susceptible to PGIA and PGIS, produced the highest levels of anti-inflammatory IL-4, proinflammatory IL-6 and IFN-γ cytokines when assayed in spleen cultures. However, there was no evidence that any of these cytokine genes (Figure 2) were expressed differentially in BALB/cJ versus BALB/cByJ colonies (data not shown). We hypothesized that BALB/c mice of the Hollister and ByJ 'low-susceptibility' colonies (with delayed onset and less severe arthritis) still might be in the initiative (proinflammatory) phase of arthritis at the end of the experiments (days 63 and 64). This was supported by the serum levels of autoantibodies to mouse PG (either IgG1 or IgG2a), which were significantly lower in animals of group III than in any other colonies (data not shown). Indeed, in supplemental experiments using age-matched females of The Jackson Laboratory's BALB/cJ and BALB/cByJ colonies or of Kingston and Hollister colonies of Charles River Laboratories, Inc. (average versus low-susceptibility animals) injected with the standard dose of 100 μg of PG protein on day 42 (third immunization), these particular midterm differences disappeared (data not shown).

### Relationship between immune responses and clinical features

Next, we compared arthritis- and spondylitis-'specific' immune markers between the three groups of the clinical phenotypes (Table 2). We compared serum antibody, cytokine, and antigen-specific *in vitro *T-cell responses of 154 arthritic animals with 24 immunized as-yet-non-arthritic mice (Table 2). The incidence of PGIA in the three major groups was as follows: 98% in group I, 85% in group II, and 40% in group III. Although there was a trend, we found that none of the *in vitro*-measured T-cell activation markers (antigen-specific T-cell proliferation and cytokine production) correlated significantly with the clinical phenotype (severity) or histological results of arthritis. In contrast, IgG1 and IgG2a (auto)antibodies were significantly higher in arthritic than in non-arthritic animals (Table 2).

**Table 2:** Immunological differences between arthritic and non-arthritic animals as well as mice with or without spondylitis

Measured parameters	Arthritic animals (n = 154)	Non-arthritic animals (n = 24)	Mice with spondylitis (n = 80)	Mice without spondylitis (n = 47)
*In vitro *T-cell proliferation, SI	2.96 ± 0.07	3.25 ± 0.13	3.00 ± 0.09	3.00 ± 0.14
*In vitro *IL-2 production (CTLL-2), SI	2.67 ± 0.07	2.73 ± 0.15	2.68 ± 0.10	2.66 ± 0.11
*In vitro *IL-4 production, ng/10^6 ^cells	2.65 ± 0.14	2.91 ± 0.34	2.79 ± 0.22	2.39 ± 0.18
*In vitro *IL-6 production, ng/10^6 ^cells	1.83 ± 0.01	2.17 ± 0.33	1.88 ± 0.14	1.86 ± 0.19
*In vitro *IFN-γ production, ng/10^6 ^cells	8.06 ± 0.28	8.50 ± 1.07	7.61 ± 0.37	9.56 ± 0.65^a^
*In vitro *TNF-α production, ng/10^6 ^cells	0.74 ± 0.01	0.77 ± 0.02	0.74 ± 0.01	0.75 ± 0.02
Serum IL-4, pg/mL	15.29 ± 2.41	22.80 ± 5.92	15.52 ± 3.32	18.49 ± 4.46
Serum IL-6, pg/mL	166.45 ± 20.21	106.18 ± 26.97	97.59 ± 14.94	65.05 ± 20.61
Serum IL-1β, pg/mL	96.36 ± 11.55	41.70 ± 11.29	163.78 ± 27.72	143.10 ± 31.16
Serum IFN-γ, pg/mL	26.14 ± 2.62	34.59 ± 6.27	25.05 ± 3.65	33.49 ± 4.55
IgG1 antibodies to human PG, mg/mL	12.75 ± 0.57^b^	8.30 ± 0.94	23.33 ± 0.78^b^	8.85 ± 0.86
IgG2a antibodies to human PG, mg/mL	1.36 ± 0.14^a^	0.72 ± 0.29	1.44 ± 0.21^a^	0.82 ± 0.17
IgG1 antibodies to PG, μg/mL	172.55 ± 13.47^b^	76.41 ± 10.56	174.99 ± 18.95^a^	116.68 ± 17.7
IgG2a antibodies to PG, μg/mL	68.01 ± 5.68^b^	24.79 ± 6.76	72.10 ± 8.18^b^	34.57 ± 6.16

All animals were immunized simultaneously with human cartilage proteoglycan in dimethyldioctadecyl-ammonium bromide. A total of three injections were given at 3-week intervals, and mice were sacrificed 3 weeks after the third injection (on days 63 and 64) when all *in vitro *assays were performed. Values represent mean ± standard error of the mean. Histological analysis was performed, and each inflamed intervertebral disc (IVD) was scored from '0' to '8' as described in Materials and methods. Positive (spondyloarthopathic) animals were combined if at least one IVD was affected with inflammation. Superscript letters indicate the level of significance (^a^*P *< 0.05 and ^b^*P *< 0.01) between arthritic and non-arthritic animals or between mice with or without spondylitis. IFN-γ, interferon-gamma; IL, interleukin; PG, proteoglycan; SI, stimulation index; TNF-α, tumor necrosis factor-alpha.

We also compared the *in vitro *antigen (PG)-specific T-cell responses and serum antibody levels in mice having (n = 80) or not having (n = 47) spondyloarthropathy. PG-stimulated spleen cell cultures expressed significantly more IFN-γ in mice without spondyloarthropathy than those that already had spine inflammation (Table 2). In contrast, all antibody isotypes (either to the immunizing human or autoantibodies to mouse cartilage PG) were significantly higher in the spondyloarthopathic animals than in those having no spondylitis (Table 2).

### Microarray results

Certain genetic differences between colonies of the same murine strain have already been analyzed (for example, The Jackson Laboratory detected 492 SNPs between BALB/cJ and BALB/cByJ colonies, two sublines that were separated about 73 years ago) [17]. Therefore, in one of our 'prototype' experiments, we compared the gene expression profile of splenocytes of these two colonies prior to, and then 12 days after, the first PG injection, when the initial immune responses are detectable but there is no arthritis. Figure 2 shows the results of the analysis of 12 microarrays using three animals in each group. All samples passed all quality control tests, and 36,816 probe sets were analyzed. As shown in the hierarchical clusterization panel, a total of 77 genes were expressed at significantly different levels between naive and immunized BALB/cJ and BALB/cByJ age-matched female mice. Twenty-three genes showed greater than twofold differences (Figure 2), and 11 of the 77 genes were described as immune response genes or associated with arthritis (Additional data file 1) [30-65]. When we compared the 77 genes expressed significantly in naïve and immunized mice, 69 were specific for naïve and only 8 genes were associated with the immunization (Figure 2) (Gene Expression Omnibus accession number [GEO:GSE13730] and National Center for Biotechnology Information tracking system number 15549466).

## Discussion

Although female BALB/c mice are close to 100% susceptible to PG-induced arthritis after three consecutive immunizations with human cartilage PG aggrecan [7,18,66], we found significant differences in arthritis severity, onset, and progression among the inbred colonies. Our findings, however, do not indicate that animals in group III acquired resistance; rather, these mice showed a tendency to develop arthritis, but they needed a longer period of time, a higher dose of antigen, or an additional (fourth) injection of human PG. Similar results were found when F2 hybrid mice of susceptible BALB/c and resistant strains were immunized and tested for arthritis- or spondyloarthropathy-associated QTLs using the same antigen, immunization protocol, and scoring system (visual and histology) and when MHC- and age-matched animals were housed in the same room, occasionally for more than half a year [9,11,23,24,29,66]. These genome-wide screening studies explored overlapping QTLs in different genetic combinations between high- or low-susceptibility F2 hybrids, indicating that different combinations of genes may affect disease onset and/or severity [9,24,29]. In this comparative study, differences among different colonies suggest that either as-yet-unknown genetic factors or differences in transforming environmental effects at the site of origin and/or our animal facility (although both are pathogen-free) caused these unexpected findings. Some of the most important environmental factors are the normal intestinal microbial flora and various bacterial cell wall components (for example, peptidoglycans) [67], which may affect the phenotype (onset and/or severity) of arthritis. Although a variation in the composition of normal bacterial flora can explain some of our findings, we have not had a chance yet to investigate intestinal flora-related differences in detail in the 11 BALB/c colonies.

Certain BALB/c substrains are known for the production of plasmacytoma in response to mineral oil injection [68], which generated a myeloma cell line (Sp2/0.Ag.14), a fusion partner with lymphoblasts routinely used in monoclonal antibody technology [69]. Moreover, though not frequently (in less than 2% of retired breeder female BALB/c mice and, so far, only in the NCI/Kingston colony), we observed spontaneous arthritis with less or more extensive synovitis and inflammation (T.T. Glant and K. Mikecz, unpublished observation), occasionally associated with cartilage erosion in small peripheral joints, which are histology features that were indistinguishable from those seen in PGIA (unpublished observation).

Although the dominant genetic factor is the MHC in both RA and PGIA, the MHC alone is insufficient to affect arthritis susceptibility and severity (for example, in H-2d DBA/2 mice) [9]. Two 'Q' subloci (Q6 and Q8) were expressed at a significantly higher level in BALB/cJ mice than in BALB/cByJ mice (Figure 2 and Additional data file 1), which might contribute to the earlier onset or more severe arthritis in BALB/cJ mice, but none of these subloci was associated with the immunized state (in Figure 2, see naïve versus immunized pairwise comparisons of the two subcolonies). Another critical factor in the pathogenesis of PGIA is the non-MHC genetic component (reviewed in [9,70]). The first albino mouse was found by a pet dealer in Ohio in 1913 [71]. Brothers and sisters were systematically mated and an inbred colony was established in 1920 [71].

The original BALB/c colony was separated in 1935. One of these colonies was maintained by G. Snell at The Jackson Laboratory (BALB/c J), and the other was maintained by H.B. Andervont (BALB/c AnN) and then transferred to the National Institutes of Health (NIH) (Bethesda, MD, USA) in 1951 [72]. All other BALB/c colonies are derived from these two ancient ancestors. Charles River Laboratories, Inc., started breeding BALB/c mice in 1974 (mice from NIH), Harlan Laboratories, Inc., (Indianapolis, IN, USA) in 1986 (mice from NIH), and Taconic Farms, Inc. (Hudson, NY, USA) in 1988 (mice were purchased from the NIH). It is also relevant to note that, except for one BALB/c colony (Hollister, CA, USA), all of the distributors are located on the East Coast or in the Midwest regions of the US. During their 88-year history, inbred BALB/c colonies have been exposed to various environmental effects (moving to another location, repopulation from other colonies due to fire, and so on). Although the companies ensure the genetic homogeneity of the colonies by applying strict breeding and maintenance rules, differences among the colonies do occur.

Historically, the BALB/cJ colony represents the original BALB/c mice of The Jackson Laboratory (maintained since 1935), whereas the BALB/cByJ mice were inherited from the NIH and the breeding stock was transferred to The Jackson Laboratory in 1967, when D.W. Bailey joined the company. The two colonies have been maintained separately and represent the pedigree of the two original BALB/c lines (The Jackson Laboratory versus NIH). Therefore, the 492 SNPs [17] and the 77 differentially expressed genes (Figure 2) of the two colonies attest to the dynamic flexibility of the mammalian (mouse) genome, which keeps changing despite being exposed to comparable environmental conditions.

Thirty-three of the 492 SNPs and 11 genes (labeled in yellow in Additional data file 1) of the 77 differentially expressed genes in BALB/cJ and BALB/cByJ mice are related to immune regulatory functions, which in turn may affect arthritis onset, severity, and susceptibility. Although most of the SNPs are present in intron sequences, some of them may have an effect on exon splicing. However, the differences in the phenotypes can hardly be explained by the SNPs and the few immunoregulatory genes that are expressed differentially in both naïve and immunized BALB/cJ and BALB/cByJ mice. Similarly, 8 of the 77 genes showed differential expression (either upregulation or downregulation) in response to immunization (Figure 2, labeled with the '†' symbol). Although we can speculate that these genes are involved in arthritis severity or onset, probably none of them is responsible for susceptibility.

An important observation was the inflammation around the IVD in arthritic BALB/c mice, which is found in up to 60% of patients with ankylosing spondylitis when examined by magnetic resonance imaging [73]. The nucleus pulposus of IVDs is composed mostly of hyaluronan and 'cartilage-specific' PG aggrecan, and the core protein of the human aggrecan molecule has over 100 predicted and at least 27 confirmed T-cell epitopes in BALB/c mice [9,74]. A number of these epitopes have been reported as dominant/arthritogenic in wild-type or humanized BALB/c mice [74-78] and are possibly involved in immune reactions to IVD components. The immune attack, characterized by a predominantly lymphocytic infiltration around the IVD in the early phase of the spondylitis [7-9], is most likely elicited by cross-recognition of IVD PG in mice immunized with human PG. Spondyloarthropathy has a progressive character and shows a correlation with the onset and progression of peripheral arthritis, although PGIA and PGIS are two independent diseases [11], as we have shown, and different genes in different QTLs control PGIA [9] and PGIS [29]. Interestingly, although inflammatory (autoimmune) spondyloarthropathy occurs only in BALB/c and C3H mice [7,8,11], spontaneous or experimentally induced disc degeneration has been reported in numerous animal models [79-82]. Autoimmune mechanisms are thought to play a major role only in HLA-B27 transgenic rodents [83-85] and in PGIA [7,9].

We expected to find robust T- and B-cell responses *in vitro *in antigen (PG)-stimulated spleen cell cultures of arthritic mice because RA is thought to be a T cell-dependent and B cell-mediated disease [86]. Due to the intense involvement of various lymphoid organs (spleen and lymph nodes) in the regulation of immune responses, the serum cytokine levels may represent a momentary status rather than a general level of *in vivo *activation [87]. In this respect, it is not surprising that we could not find significant correlations between clinical or histological findings and serum cytokine levels. On the other hand, when we analyzed and compared the results of individual animals with or without arthritis or spondylitis at the end of the observation period (that is, without pooling animals within a group [colony]), significant correlations were found (Table 2). For example, significantly higher levels of heteroantibodies and autoantibodies to cartilage PG were measured in the sera of arthritic and spondyloarthopathic animals than in as-yet-non-affected cage-mates (Table 2). An example of negative correlation was found when we compared PG-specific *in vitro *IFN-γ production by spleen cells in animals with and without spondylitis (Table 2), perhaps suggesting that Th1 T-cell activation was still restricted to the lymphoid organs before the immune attack against the spine occurred. Similar differences and/or negative-positive correlations, though at lower levels, were found when other markers were compared with the clinical phenotype.

Both RA and PGIA require T cells and B cells (or autoantibodies), in which the autoimmune attack culminates in the inflammatory destruction of peripheral joints. A number of similarities between RA and PGIA suggest that certain as-yet-unknown alterations of the immune system exist in both humans and mice. As a continuation of the experiments presented in this study, we are comparing gene expression in various lymphoid organs, and joint tissues of representative colonies at different time points after immunization, and correlating these results with clinical phenotypes of arthritis and spondylitis as well as with the results of our earlier genome-wide studies [9].

## Conclusion

The MHC (H-2d) and non-MHC components of the genetic background make the BALB/c strain highly susceptible to inflammatory arthritis and spondylitis. Although BALB/c colonies uniformly develop PGIA (> 95%) and PGIS (> 80%) in response to immunization with human cartilage PG aggrecan, even in the absence of mycobacterial components (that is, without the use of Freund's complete adjuvant), there are significant differences among BALB/c colonies maintained even by the same vendor at different locations or when the 'subcolonies' were separated several decades ago. Technically, among the BALB/c colonies tested so far, we have not found a PGIA- or PGIS-resistant colony, but the 'level of susceptibility' is different among them. This may be a critical question when laboratories use different colonies to induce other diseases, PGIA or PGIS, or when transgenic/gene-deficient mice in 'different' BALB/c backgrounds are compared with control wild-type BALB/c animals. Although this observation may be 'specific' for BALB/c colonies, or PGIA and PGIS, this might not be a correct conclusion. A mutation in critical genes may dramatically affect cell function(s), and the result of the mutation is then designated as a 'new phenotype'. However, mutations in inbred colonies occur frequently (for example, C5 deficiency in DBA/2 mice [88,89], in the cytoplasmic domain of Toll-like receptor-4 of The Jackson Laboratory's C3H/HeJ colony [90], and in the *Ptpn6 *gene of motheaten mice [91,92], and so on). Relatively small or as-yet-unidentified mutations in the genome may significantly affect disease susceptibility or eventually a series of physiological/pathophysiological functions, preferentially leading to incorrect conclusions.

Genetic components are major players in the development of PGIA, and our genome-wide studies explored close to 30 different loci (12 corresponding to human RA susceptibility loci identified in familial studies) [9]. Here, we present the results of a systemic age- and gender-matched comparative study using 11 substrains/colonies of BALB/c mice. With a suboptimal dose of arthritogenic cartilage PG, significant differences were found in arthritis susceptibility among colonies. Although no single gene or 'biomarker' that could account for these differences has been identified, the large number of SNPs in two sister colonies (The Jackson Laboratory's BALB/cJ and BALB/cByJ) separated about 70 years ago and the corresponding microarray results indicate that, indeed, a single or a limited number of mutations may dramatically affect the clinical phenotype of arthritis in BALB/c mice. The differences identified among colonies may help us to target disease-affecting gene(s) and may become nearly as valuable a tool as subcongenic approaches. The results of our study may serve as a direction toward a more accurate selection of disease-controlling genes from previously identified QTLs, especially from those that are shared in RA and corresponding animal models.

## Abbreviations

DDA: dimethyldioctadecyl-ammonium bromide; ELISA: enzyme-linked immunosorbent assay; IFN-γ: interferon-gamma; IL: interleukin; IVD: intervertebral disc; MHC: major histocompatibility complex; NCI: National Cancer Institute (Bethesda, MD, USA); NIH: National Institutes of Health (Bethesda, MD, USA); PG: proteoglycan; PGIA: proteoglycan-induced arthritis; PGIS: proteoglycan-induced spondylitis; QTL: quantitative trait locus; RA: rheumatoid arthritis; RUMC: Rush University Medical Center (Chicago, IL, USA); SNP: single-nucleotide polymorphism.

## Competing interests

The authors declare that they have no competing interests.

## Authors' contributions

HLR and TTG made the first observations for the differences between BALB/c (NCI, Hollister, and Jackson) colonies. These preliminary results led to the current study. BF carried out the most significant part of the research in Chicago, was involved in manuscript writing, and helped to perform the statistical analysis, to score animals, and to collect and pulverize human cartilage samples. FB performed T-cell separation and tissue culture and helped to perform the statistical analysis. BF and FB contributed equally to this work. OT isolated, purified, and prepared RNA for microarray hybridization. AL and SML helped to perform the statistical analysis. BT controlled and supervised animals on a daily basis. GH helped to score animals and to collect and pulverize human cartilage samples. GN and AM helped to measure serum cytokines and antibodies. TTG isolated and purified PG antigen for immunization, designed and coordinated all experiments, and prepared the final version of the manuscript. AF and KM helped to coordinate and supervise the immunizations and contributed to data selection, interpretation of results, and manuscript preparation. All authors read and approved the final manuscript.

## Supplementary Material

Additional data file 1 Word file listing 77 genes (including 14 genes with yet unknown functions) expressed differently at significant levels in spleen cells of naive and proteoglycan-immunized (non-arthritic) mice of BALB/cJ and BALB/cByJ colonies (as shown in Fig 2 as hierarchical clusterization). Four-group cross-comparison were used: naive BALB/cJ vs. immunized BALB/cJ, naive BALB/cByJ vs. immunized BALB/cByJ, naive BALB/cJ vs. naïve BALB/cByJ and immunized BALB/cJ vs. immunized BALB/cByJ). This file contains probe set identification numbers (Affymetrix), gene symbols, names and their chromosome localization, Ensembl numbers, p values, and brief description of gene function (if known). Genes with unknown functions are highlighted in blue, and genes having immuno-regulatory function in yellow. The corresponding references of these immuno-regulatory genes are [30-65]. Original data (.cel) files are submitted to Gene Expression Omnibus (Accession Number [GEO:GSE13730]; NCBI tracking system number 15549466). Click here for file
