# Supplementary material for: BALB/c mice genetically susceptible to proteoglycan-induced arthritis and spondylitis show colony-dependent differences in disease penetrance
Source: Arthritis Res Ther. 2009 Feb 16;11(1):R21. doi: 10.1186/ar2613 (PMC2688253; doi:10.1186/ar2613)
Supplement: Supplementary file 1 [file ar2613-S1.doc]

Row number (Fig 2)	Probe Set ID	Gene Symbol	Gene Title	Ensembl	Entrez Gene	Corrected p-value	Chromosome Number (Assembly)	Chromosome Start Index (Assembly) (kb)	Chromosome End Index (Assembly) (kb)	Function / biological process / cellular localization	Ref.
1	1415961_at	Mrpl48	mitochondrial ribosomal protein	ENSMUSG[illegible]	94067	0.045	chr19	45.079	45.081	mitochondrial ribosome 39S	
2	1438116_s_at	Slc9a3r1	solute carrier family 9 (sodium/hydrogen exchanger), member 3 regulator	ENSMUSG[illegible]	26941	0.029	chr11	115.042	115.062	WNT receptor signaling pathway, camp-mediated signaling, cellular phosphorylation	
3	1449001_at	Ivd	isovaleryl coenzyme A dehydrogenase	ENSMUSG[illegible]	56357	0.032	chr2	118.688	118.709	oxidoreductase activity, mitochondrion	
4	1452846_at	Tp53inp2	transformation related protein 53 inducible nuclear protein	ENSMUSG[illegible]	68728	0.047	chr2	155.208	155.216	nucleus, developing brain and spinal cord, sensory and motor neuron cells in peripheral nervous	
5	1449111_a_at	Grb2	growth factor receptor bound protein 2	ENSMUSG[illegible]	14784	0.047	chr4	101.546	101.5[illegible]	[illegible] MAPKKK cascade, Ras protein signal transduction, cell	
6	1419209_at	Cxcl1	chemokine (C-X-C motif) ligand	ENSMUSG[illegible]	14825	0.036	chr5	91.320	91.322	chemokine/cytokine/growth factor activity, immune/inflammatory	57-61
7	1429947_a_at	Zbp1	Z-DNA binding protein 1	ENSMUSG[illegible]	58203	0.025	chr2	173.029	173.044	DNA mediated innate immunity	62-64
8	1420577_at	*Aicda	activation-induced cytidine deaminase	ENSMUSG[illegible]	11628	0.035	chr6	122.504	122.514	isotype switching, mRNA processing, EXO2 mouse - spontaneous arthritis	65-69
9	1452242_at	*Cep55	centrosomal protein 55	ENSMUSG[illegible]	74107	0.012	chr19	38.130	38.149	cell cycle/division, mitosis	
10	1428176_at	*Edg5	endothelial differentiation, sphingolipid G-protein-coupled receptor	ENSMUSG[illegible]	14739	0.021	chr9	20.770	20.775	sphingosine-1-phosphate receptor S1P(2) antagonist	70
11	1452871_at	*Neil1	nei endonuclease VIII-like 1 (E.	ENSMUSG[illegible]	72774	0.021	chr9	56.991	56.996	DNA repair	
12	1451706_at	*Cilp	cartilage intermediate layer protein, nucleotide	ENSMUSG[illegible]	214425	0.029	chr9	65.122	65.128	extracellular matrix	71-73
13	1455216_a_at	Syt11	synaptotagmin XI	ENSMUSG[illegible]	229521	0.026	chr3	88.540	88.556	synapse, cell junction, membrane transport	
14	1431182_at	*Hspa8	heat shock protein 8 /// hypothetical LOC634852 /// similar to heat shock protein 8 /// similar to heat shock protein 70	ENSMUSG[illegible]	15481	0.046	chr9	40.608	40.613	chaperone, protein binding, stress response, cell cycle regulation	
15	1420935_s_at	Cpsf2	cleavage and polyadenylation specific factor 2	ENSMUSG[illegible]	51786	0.029	chr12	103.214	103.244	RNA binding, mRNA	
16	1416213_s_at	Surf4	surfeit gene 4	ENSMUSG[illegible]	20932	0.012	chr2	26.776	26.788	endoplasmic reticulum	
17	1434114_at	Rexo4	REX4, RNA exonuclease 4 homolog (S.	ENSMUSG[illegible]	227656	0.039	chr2	26.809	26.812	XPMC2 prevents mitotic catastrophe 2	
18	1418909_at	*Pira2	paired-Ig-like receptor A2	ENSMUSG[illegible]	18725	0.032	chr7	3.664	3.691	expressed by B cells and myeloid cells	74
19	1441835_at	*1441835_at	Transcribed locus	-	-	<0.001	chr18	86.674	86.675	unknown	
20	1456283_at	*Neto1	neuropilin (NRP) and tolloid (TLL)-like	ENSMUSG[illegible]	246317	<0.001	chr18	86.571	86.615	neuronal, brain	
21	1456719_at	*1456719_at	Transcribed locus, moderately similar to NP_001013091.1 BTB (POZ) domain containing 9 [Rattus	ENSMUSG[illegible]	1000[illegible]	<0.001	chr17	31.060	31.062	unknown	
22	1436070_at	*Glo1	glyoxalase 1	ENSMUSG[illegible]	109801	<0.001	chr17	30.725	30.737	cytosol, conversion of a cocarboxylic... into the corresponding α-hydroxy acid using glutathione (GSH) as cofactor	75,76
23	1455210_at	*Zhx2	zinc fingers and homeoboxes 2	ENSMUSG[illegible]	387609	<0.001	chr15	57.610	57.671	transcription factor, regulation of transcription	
24	1429880_at	*0710001D07Rik	RIKEN cDNA 0710001D07	-	67619	<0.001	chr17	30.713	30.714	unknown	
25	1431007_at	*2210404O11Rik	RIKEN cDNA 2210404O11 gene /// RIKEN cDNA 9030025P20 gene /// hypothetical protein	ENSMUSG[illegible]	1000[illegible]	0.001	chr17	15.118	15.125	unknown	
26	1438598_at	Rttn	rotatin	ENSMUSG[illegible]	246102	0.008	chr18	89.300	89.380	development, left-right symmetry	
27	1452700_s_at	Ndufa2	NADH dehydrogenase (ubiquinone) 1 alpha subcomplex, 2	ENSMUSG[illegible]	68091	0.035	chr7	3.569	3.572	mitochondrion, electron transport	
28	1422718_at	Ap3s2	adaptor-related protein complex 3, sigma 2	ENSMUSG[illegible]	11778	0.022	chr7	87.025	87.066	Golgi, protein transport	
29	1427496_a_at	*Dnahc8	dynein, axonemal, heavy chain 8	ENSMUSG[illegible]	13417	0.027	chr17	30.764	31.012	microtubule motor, microtubule based	
30	1436419_a_at	*1700097N02Rik	RIKEN cDNA 1700097N02	-	67522	0.022	chr17	30.759	30.761	unknown	
31	1425124_s_at	Rad54l	RAD54 like (S. cerevisiae)	ENSMUSG[illegible]	19366	0.020	chr4	115.787	115.796	DNA repair, chromosome	77,78
32	1449429_at	Fkbp1b	FK506 binding protein 1b	ENSMUSG[illegible]	14226	0.016	chr12	4.840	4.848	FK506 binding, T cell proliferation, Ca signaling, pancreas, cardiac muscle	
33	1438962_s_at	Tfpt	TCF3 (E2A) fusion partner	ENSMUSG[illegible]	69714	0.035	chr7	3.572	3.572	apoptosis induction	
34	1417131_at	Cdc25a	cell division cycle 25 homolog A (S.	ENSMUSG[illegible]	12530	0.012	chr9	109.778	109.796	cell cycle/division, mitosis	
35	1428582_a_at	Anp32a	acidic (leucine-rich) nuclear phosphoprotein 32 family, member	ENSMUSG[illegible]	11737	0.003	chr9	[illegible]	[illegible]	phosphatase inhibitor	
36	1429285_s_at	Trip13	thyroid hormone receptor interactor 13	ENSMUSG[illegible]	69716	0.023	chr13	74.050	74.075	meiosis, DNA repair	
37	1421622_at	Tcte3	t-complex-associated testis expressed 3 /// t-complex-associated testis expressed 3-like	ENSMUSG[illegible]	1000[illegible]	0.035	chr17	15.128	15.147	unknown	
38	1433845_a_at	6430706D22Rik	RIKEN cDNA 6430706D22 gene /// hypothetical protein	ENSMUSG[illegible]	1000[illegible]	0.022	chr1	90.160	90.170	unknown	
39	1438571_at	Bub1	budding uninhibited by benzimidazoles 1 homolog (S.	ENSMUSG[illegible]	12235	0.029	chr2	127.626	127.657	cell cycle/division, mitosis, chromosome	
40	1419596_at	Arid2	RIKEN cDNA 1700124K17 gene /// AT rich interactive domain 2 (Arid-rfx like)	ENSMUSG[illegible]	[illegible]	0.041	chr15	96.232	96.3[illegible]	transcription	
41	1419951_at	Lman1	lectin, mannose-binding, 1	ENSMUSG[illegible]	70361	0.050	chr18	66.140	66.141	sugar binding, ER, Golgi, protein	
42	1448279_at	Pot1a	protection of telomeres 1A	ENSMUSG[illegible]	101185	0.029	chr6	25.694	25.759	telomere maintenance	
43	1427500_at	Zranb1	zinc finger, RAN-binding domain containing	ENSMUSG[illegible]	360216	0.022	chr7	140.165	140.177	unknown	
44	1447052_at	Clybl	citrate lyase beta like	ENSMUSG[illegible]	69634	0.046	chr14	122.644	122.644	cellular aromatic compound metabolic process	
45	1458458_at	Tmem57	Transmembrane protein 57	ENSMUSG[illegible]	66146	0.050	chr4	134.366	134.396	neuronal	
46	1456447_at	*Rpl18	ribosomal protein L18	ENSMUSG[illegible]	19899	0.046	chr7	52.976	52.978	translation, cytoplasm	
47	1438501_at	*Atg2b	ATG2 autophagy related 2 homolog B (S.	ENSMUSG[illegible]	76559	0.026	chr12	106.852	106.853	unknown	
48	1442015_at	Ccdc79	coiled-coil domain containing	ENSMUSG[illegible]	320092	0.020	chr8	106.072	106.097	DNA binding	
49	1446606_at	LOC625175	region containing RIKEN cDNA 1700028N10 gene; hypothetical protein	-	625175	0.022	chr5	152.184	152.185	unknown	
50	1460409_at	*Irak1	interleukin-1 receptor-associated kinase 1	ENSMUSG[illegible]	16179	0.050	chrX	71.259	71.269	cytokine/chemokine-mediated signaling, innate immunity, LPS	79-81
51	1431062_at	*Ganc	glucosidase, alpha; neutral C	ENSMUSG[illegible]	76051	0.040	chr2	120.272	120.287	glucose metabolism	
52	1452700_at	Def6	differentially expressed in FDCP 6	ENSMUSG[illegible]	23853	0.023	chr17	28.362	28.368	plays a role in Th2 development and/or activation, perhaps by interfering with ZAP-70	82-85
53	1416762_at	S100a10	S100 calcium binding protein A10	ENSMUSG[illegible]	20194	0.046	chr3	93.359	93.368	[illegible] adapter protein, modulator, neuronal	86
54	1438902_at	H2-Q8	histocompatibility 2, Q region locus	ENSMUSG[illegible]	15019	0.013	chr17	35.551	35.555	MHC-I like, antigen	
55	1419620_at	*St6galnac5	ST6 (alpha-N-acetyl-neuraminyl-2,3-beta-galactosyl-1,3)-N-acetylgalactosaminide alpha-2,6-sialyltransferase	ENSMUSG[illegible]	26938	0.022	chr3	152.483	152.645	[illegible] acid glycosylation	
56	1425242_at	*Cd207	CD 207 antigen	ENSMUSG[illegible]	246278	0.012	chr6	83.621	83.628	langerin, Langerhans cell specific c-type lectin	
57	1429809_a_at	*Arl2bp	ADP-ribosylation factor-like 2 binding	ENSMUSG[illegible]	107566	<0.001	chr8	97.181	97.187	small GTPase regulator activity	
58	1460247_at	2410001C21Rik	RIKEN cDNA 2410001C21	ENSMUSG[illegible]	66404	<0.001	chr2	172.256	172.265	unknown	
59	1460216_at	Acads	acyl-Coenzyme A dehydrogenase, short chain	ENSMUSG[illegible]	11409	<0.001	chr5	115.560	115.569	fatty acid metabolism	
60	1434818_a_at	Tcfe3	transcription factor E3	ENSMUSG[illegible]	209446	<0.001	chrX	7.344	7.352	regulation of transcription	
61	1438571_at	*1438571_at	Transcribed locus	-	-	<0.001	chr4	138.447	138.448	unknown	
62	1451644_a_at	*H2-Q6	histocompatibility 2, Q region locus 1 /// RIKEN cDNA 0610037M15 gene /// histocompatibility 2, Q region	ENSMUSG[illegible]	110557	0.003	chr17	35.517	35.521	MHC-I like, antigen	
63	1449452_a_at	*Gp2	glycoprotein 2 (zymogen granule	ENSMUSG[illegible]	67133	0.016	chr7	126.596	126.603	GPI-anchored protein, major membrane protein present in zymogen granules of the exocrine	
64	1433946_at	Tmem181	transmembrane protein 181 /// similar to G protein-coupled receptor	ENSMUSG[illegible]	1000[illegible]	0.023	chr17	6.270	6.306	embryonic development	
65	1434726_at	Tmem146	transmembrane protein 146	ENSMUSG[illegible]	[illegible]	0.027	chr17	56.882	56.904	unknown	
66	1452101_at	*Klhl25	kelch-like 25 (Drosophila)	ENSMUSG[illegible]	207952	0.035	chr7	82.018	82.030	unknown	
67	1440075_at	*LOC552874	hypothetical LOC552874	-	552874	0.050	chr3	152.460	152.461	unknown	
68	1460283_at	BC028528	cDNA sequence BC028528	ENSMUSG[illegible]	228788	0.012	chr3	152.893	152.907	unknown	
69	1416871_at	Adam8	a disintegrin and metallopeptidase domain	ENSMUSG[illegible]	11501	0.046	chr7	147.165	147.178	cell adhesion, integrin-mediated	87-90
70	1455190_at	AA407270	expressed sequence AA407270	ENSMUSG[illegible]	102371	0.031	chr9	71.352	71.358	unknown	
71	1435040_at	Dclk1	doublecortin-like kinase 1	ENSMUSG[illegible]	13175	0.035	chr3	55.342	55.348	nervous system development	
72	1451686_at	Acly	ATP citrate lyase	ENSMUSG[illegible]	104112	0.050	chr11	100.328	100.389	acetyl-CoA biosynthesis, cytoplasm	
73	1455274_at	*1455274_at	Transcribed locus, weakly similar to XP_001375135.1 PREDICTED: similar to envelope [Monodelphis	-	-	0.032	chr7	4.340	4.340	unknown	
74	1417856_at	Relb	avian reticuloendotheliosis viral (v-rel) oncogene related B	ENSMUSG[illegible]	19698	0.050	chr7	20.118	20.135	Th1 differentiation, transcription factor	91
75	1452228_at	2700086G24Rik	RIKEN cDNA 2700086G24	-	[illegible]	0.046	chr14	64.283	64.285	unknown	
76	1451764_at	She	Src homology 2 domain-containing transforming protein	ENSMUSG[illegible]	214547	0.046	chr3	89.642	89.662	SH2 domain	92
77	1437632_at	Ankrd11	ankyrin repeat domain 11 /// hypothetical protein	ENSMUSG[illegible]	[illegible]	0.041	chr8	125.417	125.454	DNA binding, bone	

blue	Columns that contains genes with unknown functions: Probe Set ID, RIKEN ID, LOC..., AA...
<0.001	FDR-corrected p-value < 0.001
Yellow	Immunological / biological function of genes potentially related to, or associated with
* Asterisk	genes with a greater than 2 Fold Change in any one of four comparisons (naive BALB/cJ vs. imm. BALB/cJ; naive BALB/cByJ vs. imm. BALB/cByJ; naive BALB/cJ vs. naive BALB/cByJ; imm. BALB/cJ vs. imm.
†	Genes which showed significant difference in response to immunization (n=6)

Gene references list in the paper [illegible]
Reference list in Additional Data File
